# Determination of Lactones in Wines by Headspace Solid-Phase Microextraction and Gas Chromatography Coupled with Mass Spectrometry

**DOI:** 10.1155/2014/863019

**Published:** 2014-03-20

**Authors:** S. J. Pérez-Olivero, M. L. Pérez-Pont, J. E. Conde, J. P. Pérez-Trujillo

**Affiliations:** Department of Analytical Chemistry, Nutrition and Food Science, University of La Laguna, La Laguna, 38071 Tenerife, Spain

## Abstract

Application of headspace solid-phase microextraction (HS-SPME) coupled with high-resolution gas chromatographic (HRGC) analysis was studied for determining lactones in wines. Six different SPME fibers were tested, and the influence of different factors such as temperature and time of desorption, ionic strength, time of extraction, content of sugar, ethanol, tannins and anthocyanins, and pH and influence of SO_2_ were studied. The proposed HS-SPME-GC method is an appropriate technique for the quantitative analysis of *γ*-butyrolactone, *γ*-hexalactone, *trans*-whiskey lactone, *γ*-octalactone, *cis*-whiskey lactone, *γ*-nonalactone, *γ*-decalactone, *δ*-decalactone, and *γ*-undecalactone in wines. Method reproducibility and repeatability ranged between 0.6 and 5.2% for all compounds. Detection limit for *γ*-butyrolactone was 0.17 mg/L and a few *μ*g/L for the rest of the compounds. The optimized method has been applied to several wine samples.

## 1. Introduction

Among volatile components of wine, lactones and particularly the *γ*-lactones and whiskey lactones play an important role in terms of their contribution to the aroma.

*γ*-Lactones series, *δ*-lactones, and whiskey lactones are the most abundant lactones in wines and the more sensory important lactones.

Lactone smell is usually described as “fruity” or “coconut-like, fruity” (*γ*-hexalactone); “coconut-like” (*γ*-octalactone); “peach-like, milky” (*γ*-decalactone), or “fruity, sweet floral” (*γ*-dodecalactone). These compounds are formed by cyclisation of the corresponding *γ*-hydroxycarboxylic acids [1].

Lactones are among the most important compounds contributing to the sensory characteristics of wines aged in oak wood. Whiskey lactones and some volatile phenols coming directly from wood have been recognized as important odor active compounds in Madeira wines [1, 2].

They are already present in natural oak and their content increases due to ageing. From an organoleptic point of view, they are the most important lactones extractable from oak casks. Furthermore, they have been reported as potential aging markers in Madeira wines [3, 4].

Oak species, geographical origin, silvicultural treatment of tree, and processing of wood have influence on volatile composition of barrel wood. These volatile compounds are susceptible to migrate from oak wood to wine. Although the volatile composition of wine undergoes an evolution during bottle aging, this is carried out in such a way that the most important characteristics spread into wine from wood and remain until the end of bottle aging. These conclusions emphasize the importance of species and geographical origin of oak wood in the volatile composition of wines during aging [5].

Other less studied lactones such as pantolactone and the 4-carbethoxy-*ç*-butyrolactone have been found in samples submitted to oxidative ageing specially in barrel-aged wine [6].

Chemical structure defines their sensorial and chemical properties [6, 7]. Lactones aromatic descriptors are influenced by the type of aromatic ring, functional groups, and substituent length chain [8]. Synergic effects can exist due to smell similarities [9].

*cis*-Whiskey-lactone isomer is a stronger odorant than the* trans*-isomer reflected by its olfaction threshold and is an important contribution to wine aroma [10]. The rest of lactones can influence wines like Zinfandel, Pinot Noir, Merlot, and Cabernet Sauvignon, particularly, *γ*-nonalactone and *δ*-decalactone with smell values higher than threshold [8].

*γ*-Butyrolactone is considered as an inductor of physical and psychic addictions and has been classified as psychotropic by FDA (US Food and Drug Administration) [11].

Different methods have been proposed for extraction of wine. Analytical methods for gas chromatography determination of lactones need a previous concentration step due to the low concentrations existing in wine [6, 10, 12]. However, these methodologies are not free from artefacts [13], and none of them has been optimized for a wide group of lactones.

A great number of wine aroma compounds have been characterised as lactones, many of them extracted by SPME. Published works account for particular compounds like diacetyl [14], sotolon [15], families of compounds such as sulphides and disulphides [16, 17], aldehydes [18–20], esters [21], alcohols [22], butyltin compounds [23], terpenes [24, 25], acetals [26], fatty acids [27, 28], or wide sets of different volatile compounds [29, 30].

The aim of this work was to apply the GC-MS technique combined with automatic headspace (HS) SPME to develop a new method to determine a set of lactones in wine (*γ*-butyrolactone, *γ*-hexalactone, *γ*-octalactone, whiskey-lactone, *γ*-nonalactone, *γ*-decalactone, *δ*-decalactone, and *γ*-undecalactone) and to apply the method to determine lactone content in white and red wines samples.

## 2. Experimental

### 2.1. Chemicals and Reagents

The following lactones were studied (CAS number in brackets): *γ*-butyrolactone [96-48-0], *γ*-hexalactone [695-06-7], *γ*-octalactone [104-50-7],* cis-* and* trans-*whiskey-lactones [39212-23-2], *γ*-nonalactone [104-61-0], *γ*-decalactone [706-14-9], *δ*-decalactone [705-86-2], and *γ*-undecalactone [104-67-6]. *γ*-Heptalactone [105-21-5] and 3,4-dimethylphenol [95-65-8] were used as internal standards (IS). These standards with purity above 99% were supplied by Aldrich (Steinheim, Germany, and Milwaukee, WI, USA) and Fluka (Buchs, Switzerland). Sodium chloride [7647-14-5] supplied by Merck was used to control ionic strength. Ethanol (analytical reagent grade; Merck, Darmstadt, Germany) [64-17-5] and Milli-Q water (Millipore, Bedford, USA) were used as solvents. Commercial tannins and anthocyanins were purchased from Agrovin (Alcazar de San Juan, Spain); sucrose [57-50-1], tartaric acid [87-69-4], potassium disulfite [16731-55-8], and sodium hydroxide [1310-73-2] (analytical reagent grade) were purchased from Panreac (Barcelona, Spain).

Individual stock standard solution in ethanol of *γ*-butyrolactone, *γ*-hexalactone, *γ*-heptalactone, *γ*-octalactone, *γ*-nonalactone, *γ*-decalactone, *δ*-decalactone, and *γ*-undecalactone and 3,4-dimethylphenol (5000 mg/L) and whiskey-lactone (10000 mg/L) were prepared. Concentrated synthetic wine solution containing L(+)-tartaric acid [87-69-4] (11 g/L) and ethanol (13%) was prepared and adjusted to pH 3.2 with sodium hydroxide and was used to prepare all synthetic test solutions. Stock solution of potassium disulfite (5.55 g/L) in water and tannins (25.39 g/L) with anthocyanins (127.00 g/L) in ethanol (Agrovin, Ciudad Real, Spain) were also prepared. Individual internal standard stock solution containing *γ*-heptalactone (13.12 mg/L) and 3,4-dimethylphenol (12.93 mg/L) were prepared using ethanol (13%). The rest of the solutions were prepared by mixture and dilution of these stock solutions.

All parameters have been optimized using a synthetic wine solution containing concentrations of lactones.  Table 1 shows those concentrations and the low and high level concentrations used for recoveries calculations.

Either individual stock standard solutions or real wine samples were prepared in 2 mL vials adding 0.77 mL of sample and 0.03 mL of internal standard solution. The vials were tightly capped with PTFE-lined cap and shaken for 10 min at 200 min^−1^.

### 2.2. Equipment

Regularly verified pipettes and class A volumetric flasks were used in solution preparation. A precision balance (Sartorius BP 210-S), a pH meter (WTW, pH 197-S), Milli-Q gradient A10 (Millipore), and a mechanical shaker (Selecta, Rotabit) were used in the study.

### 2.3. SPME Fibers

Six fibers coated with different stationary phases and various film thicknesses were purchased from Supelco (Bellefonte): polydimethylsiloxane 100 *μ*m (PDMS/100), carboxen-polydimethylsiloxane 75 *μ*m (CAR/PDMS), polydimethylsiloxane-divinylbenzene 65 *μ*m (PDMS/DVB), polyacrylate 85 *μ*m (PA), Carbowax-divinylbenzene 65 *μ*m (CW/DVB), and divinylbenzene-carboxen-polydimethylsiloxane 50/30 *μ*m (DVB/CAR/PDMS). All fibers were conditioned according to manufacturer recommendations.

### 2.4. Chromatography

The analyses were carried out on a 3800 GC gas chromatograph equipped with an 8200 Standalone autosampler, a 1079 split/splitless injector, and a mass spectrometry detector Saturn 2000 (Varian, Walnut Creek, CA, USA). Injections were performed in splitless mode, using a 0.75 mm I.D. liner which improved GC resolution. Ionization mode used was electronic impact.

Separations were performed using a DB-WAXETR capillary column (60 m, 0.25 mm I.D., 0.5 *μ*m film thickness) (J&W Scientific) with an injector temperature of 250°C (valid for all fibers) and an oven temperature program of 100°C (5 min), 8°C/min, 240°C, and 240°C (7.5 min). Carrier gas was helium at 2 mL/min flow. Peak identification was accomplished using retention time and experimental spectra obtained from individual standard solutions and confirmed using the NIST mass spectra database (Standard Reference Data of National Institute of Standards and Technology, USA).

The GC-MS transfer line temperature was 240°C. The MS operated in electron impact mode at 70 eV and collected data at a rate of 1.0 scans/s over a mass range of m/z 25–350. The ion source temperature was 200°C, the detector voltage was set to 1500 V, and the detector temperature was 300°C.

### 2.5. Experimental Design

Chromatographic conditions have been set using both synthetic solutions and wine samples to ensure a good chromatographic resolution and no coelution of compounds.

Extraction time and reproducibility, extraction temperature, desorption time and temperature, and ionic strength were optimized as a need of establishing basic instrumental parameters and simultaneously for selecting the appropriate SPME fiber.

The following steps are designed to reveal and correct possible matrix effect due to specific parameters such as phenolics, sugar, pH, sulphur dioxide, and ethanol.

## 3. Results and Discussion

### 3.1. Extraction Time

**Figure 1:**
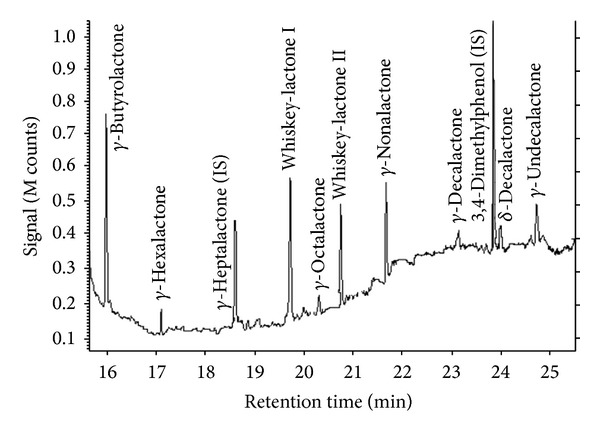
TIC chromatogram obtained with a PA fiber for a synthetic wine spiked with the different analytes.

Figure 1 shows an example chromatogram of a spiked wine sample for the chromatographic conditions cited above. It shows no peak coelution and the mixture is eluted in 25 min.

Table 2 shows retention time, molecular weight, and enthalpy of vaporization of analytes.

*γ*-Lactones show a clear direct relation between molecular weight, enthalpy of vaporization, and retention time. Compounds with higher molecular weight are less volatile and elute later.* cis-* and* trans-*Whiskey lactones elute earlier and have lower vaporization enthalpy than their C9 isomer *γ*-nonalactone probably due to structural differences, although *δ*-decalactone shows a similar behaviour as *γ*-decalactone.

Because of the kinetic nature of the extraction process, it is heavily influenced by fiber type and extraction time. Optimization of both parameters is the first step when building a microextraction method. Since the final aim of this work is to determine the analytes in sweet wines, which can have a high content in sugars (up to 200 g/L) and other many compounds, direct immersion mode leads to a rapid degradation of the fiber surface. To avoid this effect, all the studies were performed in headspace mode.

In order to establish optimal extraction parameters, the six fibers named above were studied. Experiences were made varying extraction time from 15 min to 90 min using a spiked synthetic wine.

**Figure 2:**
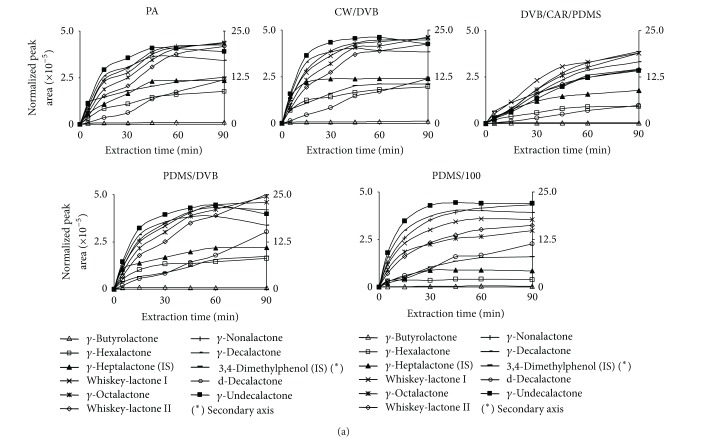
Extraction profiles of the different analytes versus extraction time for the different fibers.

Figure 2 shows normalized peak areas (absolute peak area/analyte concentration) for different analytes as a function of extraction time for the fibers studied.

CAR/PDMS fiber was immediately discarded because it offered very wide and low peaks shapes resulting in a poor peak resolution with lactone peaks overlapping between them.

As can be seen, *γ*-butyrolactone shows the lower peak area for all fibers studied. On the contrary, *γ*-undecalactone is used to show the higher peak area.

Almost all fibers show a fast initial increase in extraction during the first 15 min, and then it slows down until 45 min. After these time differences among compounds appear, some compounds like *γ*-heptalactone reach a saturation state shown as a horizontal graph line. On the contrary, some others like *γ*-octalactone, whiskey-lactone II, or *δ*-decalactone continue increasing with extraction time without reaching a saturation state. This is a general pattern for DVB/CAR/PDMS fiber and most compounds except *γ*-butyrolactone and *γ*-heptalactone. Finally, some analytes decrease in peak area at high extraction time probably due to competition for fiber active points with other compounds.

The extraction performance increases all over homologous series of n-*γ*-lactones. This is a general behaviour in all studied fibers.

PDMS fiber presents the lower extraction ability for *γ*-hexalactone, *γ*-heptalactone, *γ*-decalactone, and *δ*-decalactone; so this fiber was discarded from further optimization.

### 3.2. Extraction Reproducibility

A reproducibility study was made injecting five times a wine synthetic solution spiked with lactones (Table 3).

As can be observed, DVB/CAR/PDMS shows the worst RSD values for most compounds; so it was discarded for the rest of the studies.

Focusing on the rest of the three fibers, all of them present good overall reproducibility. Nevertheless, the value of 14.41 for *γ*-butyrolactone with PDMS/DVB fiber is bad enough for discarding this fiber.

Finally, PA and CW/DVB present a similar behaviour in terms of extraction and reproducibility; so any of them would be adequate. As CW/DVB has been discontinued by the manufacturer, PA fiber was selected as the best fiber for extracting lactones in wine samples. So, the rest of this study was done using PA fiber.

### 3.3. Extraction Temperature

Extraction temperature plays an important role in extraction but in two opposite ways. Increasing temperature produces desorption of molecules on the fiber decreasing sensibility. Simultaneously increasing temperature modifies liquid-gas equilibrium enriching gas phase with analytes [31, 32].

**Figure 3:**
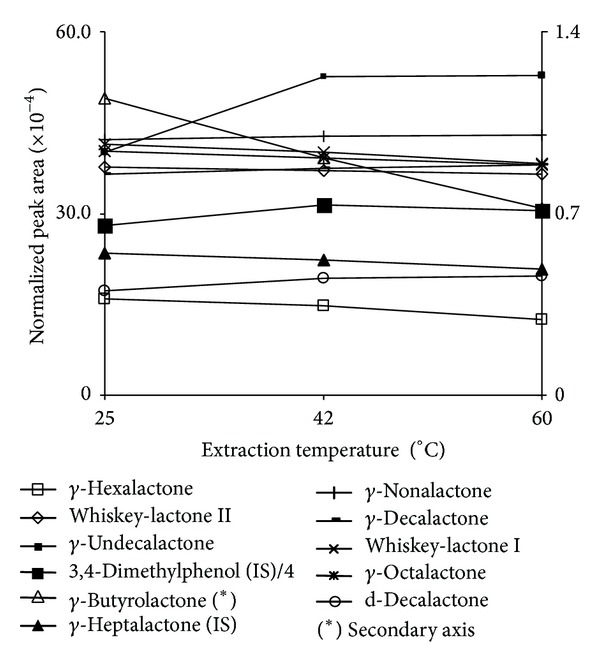
Temperature influence on the extraction of the different analytes on a PA fiber.

An extraction temperature, study temperature, was done using a synthetic wine spiked with lactones. Temperature was set to 60°C, 42°C, and 25°C using 45 min extraction time. Results of normalized peak area (peak area/concentration) versus temperatures are shown in Figure 3.

Figure 3 shows that increasing temperature leads to a decreasing extraction of both whiskey lactones and *γ*-lactones from *γ*-butyrolactone to *γ*-octalactone. The rest of the compounds show a moderate increase in extraction specially at 42°C. As lower temperatures enlarge fiber life and increasing temperature does not have a great effect, 25°C was selected as extraction temperature.

### 3.4. Desorption Time and Temperature

Desorption time and temperature were also tested within the range recommended by manufacturer. Injections were made at 250°C and 300°C desorption temperatures and 2 min, 5 min, and 10 min desorption time using the rest of selected parameters. Results are shown in Table 4.

Values of normalized peak area show increasing values with time for all analytes indicating that short desorption time leads to incomplete desorption.

On the other hand, higher temperature shows higher areas until 10 min desorption time. Taking into account that fiber life is longer at lower desorption temperatures, we selected 250°C as desorption temperature and 10 min as desorption time. Blank injections showed no memory effect in desorption for any analyte.

### 3.5. Ionic Strength

**Figure 4:**
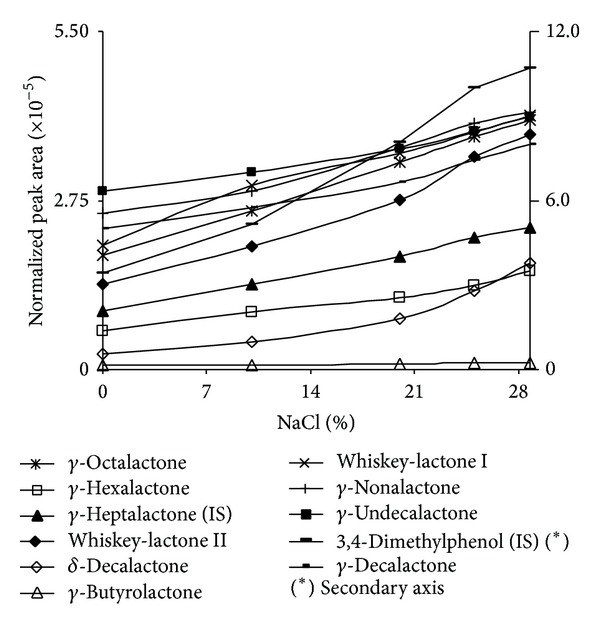
Influence of ionic strength on the extraction of the different analytes on a PA fiber.

Ionic strength affects analyte extraction, particularly those of polar character. In order to study this effect, increasing quantities of solid NaCl were added to spiked synthetic wine. Quantities of 0 mL, 80 mL, 160 mL, 200 mL, and 240 mg in 0.77 mL of sample were added to reach 0%, 10.3%, 20.7%, 25.9%, and 31.1% NaCl solutions, respectively. Results are shown in Figure 4.

Increasing ionic strength produces an increase in extraction. The best values are those obtained by saturation of NaCl and this condition is selected for further studies.

### 3.6. Phenolics, Sugar, pH, and Sulphur Dioxide Effect

Wine sample matrix has a wide variety of compounds that can affect extraction process. So it is necessary to study the effect of pH, phenolic compounds, sugar, sulphur dioxide, and ethanol content as influencing extraction process.

Polyphenol content presents wide variations in wines especially from white to red wine. An extraction study was made in order to test its influence in the process.

Synthetic wine spiked solution was prepared with tannins concentrations ranging from 0 g/L to 1 g/L and anthocyanins from 0 g/L to 5 g/L. Obtained results do not show tannins or anthocyanins influence in the extraction.

In the same way, sugar content varies widely from dry to sweet wine reaching even values higher than 200 g/L. Spiking synthetic wine with concentrations up to 200 g/L showed no influence. This is in coincidence with results reported for other compounds [18]. Wine pH usually ranges between 3 and 4 depending on grape variety and kind of wine; it is higher in red than in white wines. Different pH implies variation in dominant chemical species when acid-base properties are present. Solid-phase microextraction only extracts molecular components so ionized acids or bases remain unextracted. An extraction study was made varying pH from 3 to 4. Results showed no variation in extraction process for analytes studied.

Sulphur dioxide is a commonly used additive in wine making due to its antiseptic, antioxidant, and antioxidasic properties. Sulphur dioxide added to wine reacts with carbonyl compounds forming the so-called “combined sulphur,” especially with acetaldehyde, changing the expected concentration of free carbonyl compounds. Added sulphur dioxide quantities also change from red to white wine. To study this effect, synthetic wine solutions spiked with lactones and sodium metabisulphite ranging up to 200 mg/L were extracted. All lactones showed no influence in extraction in the range studied.

### 3.7. Ethanol Effect

**Figure 5:**
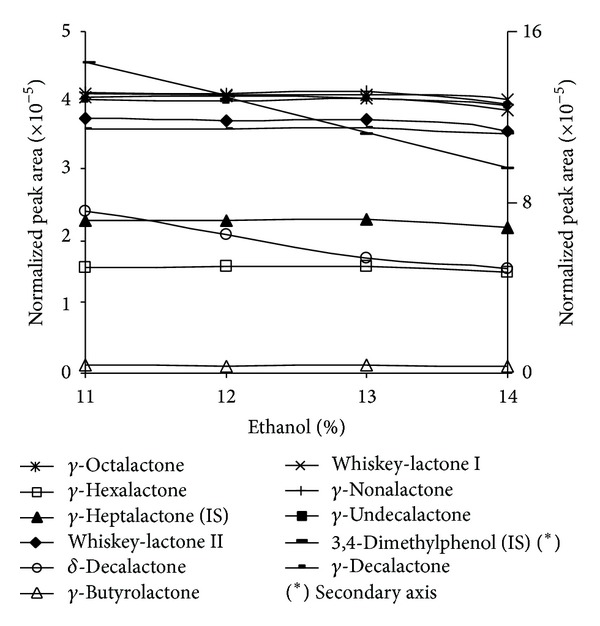
Normalized peak areas of the different analytes versus alcoholic degree on a PA fiber.

Behind water, ethanol is the major component in wines. Obviously, ethanol is extracted in fiber and effectively competes with analytes by active positions. This effect has been previously described by several authors [2, 19, 33–38]. Alcoholic degree usually ranges from 9% to 15%, but most of wines vary between 11% and 14%. So the ethanol influence was studied over synthetic wine spiked solutions with alcoholic degree in this range. Results are shown in Figure 5.

Figure 5 shows that *δ*-decalactone presented a strong decrease with increasing alcoholic degree. A similar pattern is presented by the internal standard 3,4-dimethylphenol.

The rest of lactones including the internal standard *γ*-heptalactone show no variation with ethanol increase until 13%. Higher ethanol concentrations produce a small decrease in extraction. According to these patterns, 3,4-dimethylphenol was chosen as internal standard for quantifying *δ*-decalactone and *γ*-heptalactone for the rest of analytes [16].

**Figure 6:**
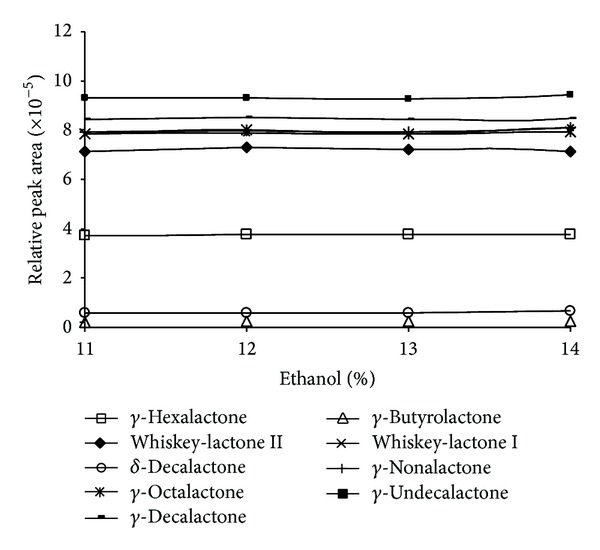
Relative peak areas versus alcoholic degree.

Figure 6 shows the relative peak areas. Relative peak areas were calculated, dividing individual standard peak area between internal standard peak areas in each chromatogram. As can be seen, relative areas appear now independent from alcoholic degree. So, internal standard quantification was selected using internal standards named above.

### 3.8. Validation

Method validation was developed in terms of linearity, detection and quantification limits, precision, and matrix effect influence.

Calibration curves were elaborated using eight synthetic wine solutions spiked with lactones, internal standards, and using the parameters selected above.  Table 5 summarizes the results. All correlation coefficients show an excellent linearity.

Detection and quantification limits were calculated as the concentration corresponding to 3 and 10 times signal/noise, respectively. Values are shown in Table 5. Most of analytes present low detection and quantification limits. The highest value is presented by *γ*-butyrolactone; but it is much lower than concentrations found in wines. In every case, detection limit is lower than odor thresholds reported.

Method repeatability and reproducibility were obtained analyzing 5 replicates of synthetic wine spiked with lactones intermediate concentration of calibration. The 5 replicates were repeated during 3 different days. Results are shown in Table 6. Both repeatability and reproducibility fall below 5% for all analytes.

Wine is a complex matrix that includes hundreds of different compounds besides those studied here. So it is necessary to perform a matrix effect study to evidence the existence of extraction interferences [7, 31–33]. In order to establish these interferences, a recovery study was realized.

Three samples of white and red wine were spiked with lactones at two different concentration levels shown in Experimental section. Results are shown in Table 7.

*γ*-Nonalactone and *δ*-decalactone are free from matrix effect showing recoveries in the range of 100 ± 10%. The most affected compounds are *γ*-butyrolactone, whiskey-lactone I, and *γ*-decalactone. Matrix effect was revealed to be similar for white and red wines.

Table 8 presents average recovery for all wines and all concentration levels. These values were used as a correction factor to obtain real concentration for analytes in real samples.

### 3.9. Analysis of Wine Samples

Optimized method was applied to 72 wine samples including white, red, and rosé wines.  Table 9 shows average concentration values (*μ*g/L) and standard deviations.

As expected, *γ*-butyrolactone is the most abundant compound for the three kinds of wine. For the rest of analytes, *γ*-hexalactone and *δ*-decalactone present the higher medium values and *γ*-octalactone, *γ*-decalactone, and *γ*-undecalactone the lower.

### 3.10. Statistical Analysis

ANOVA for these samples revealed that *γ*-hexalactone, *γ*-octalactone, and *γ*-undecalactone presented no statistically significant differences among the three kinds of wines but red wine and rosé wines presented statistically significant differences values for *γ*-butyrolactone, whiskey-lactones I and II, *γ*-nonalactone, and *δ*-decalactone. Finally, red wines had contents in *γ*-decalactone significantly higher than white wines.

## 4. Conclusions

Solid-phase microextraction is a suitable technique for determining concentrations of different lactones in wine matrix. The proposed methodology covers the range of concentrations usually found in wines with an acceptable uncertainty. The use of two internal standards corrects the influence of ethanol content. Matrix effect exists but can be corrected using both standard addition calibration and experimental correction factors, allowing the quantification of all the compounds studied using gas chromatography, mass spectrometry detection, and electronic impact.

## Figures and Tables

**Table 1:** Concentrations of synthetic solutions containing lactones.

Compound	Concentrations (*μ*g/L)		
	Low level	High level	Optimization
*γ*-Butyrolactone	14400	36200	20300
*γ*-Hexalactone	146	365	98.9
*γ*-Heptalactone			494
Whiskey-lactone I	48.3	144	100
*γ*-Octalactone	3.96	9.90	9.9
Whiskey-lactone II	48.3	144	100
*γ*-Nonalactone	19.8	59.5	100
*γ*-Decalactone	3.83	9.56	9.9
*δ*-Decalactone	124	310	96.8
*γ*-Undecalactone	1.92	4.80	9.9
3,4-Dimethylphenol			491

**Table 2:** Retention time, molecular weight and enthalpy of vaporization [31, 32].

Compound	Retention time (min)	Molecular weight (g/mol)	Δ*H*_*V*_ (KJ/mol)
*γ*-Butyrolactone	15.97	86.04	54.40 ± 0.40
*γ*-Hexalactone	17.09	114.14	57.20 ± 0.30
*γ*-Heptalactone (IS)	18.60	128.17	62.30 ± 0.30
Whiskey-lactone I (*trans*-whiskey-lactone)	19.73	156.22	48.36
*γ*-Octalactone	20.31	142.20	66.46 ± 0.40
Whiskey-lactone II (*cis*-whiskey-lactone)	20.74	156.22	48.36
*γ*-Nonalactone	21.67	156.22	70.30 ± 0.20
*γ*-Decalactone	23.14	170.25	75.60 ± 0.30
3,4-Dimethylphenol (IS)	23.86	122.16	85.70 ± 0.10***
*δ*-Decalactone	24.01	170.25	74.20 ± 0.25
*γ*-Undecalactone	24.73	184.28	79.51 ± 0.40

***Enthalpy of sublimation.

**Table 3:** RSD (%) (*n* = 5) of the relative areas of studied compounds obtained with studied fibers.

Compound	PA	DVB/CAR/PDMS	PDMS/DVB	CW/DVB
*γ*-Butyrolactone	8.14	36.43	14.41	8.47
*γ*-Hexalactone	8.24	11.76	6.56	8.94
Whiskey-lactone I	1.26	31.44	1.47	4.33
*γ*-Octalactone	1.72	26.08	2.45	2.56
Whiskey-lactone II	2.19	27.04	3.39	4.27
*γ*-Nonalactone	1.40	46.96	1.80	0.56
*γ*-Decalactone	2.45	58.36	4.40	3.24
*δ*-Decalactone*	2.91	16.77	5.23	3.71
*γ*-Undecalactone	2.78	62.05	3.96	3.37

*3,4-Dimethylphenol as IS.

**Table 4:** Normalized peak areas at different temperatures and desorption time.

Compound	250°C			300°C		
	2 min	5 min	10 min	2 min	5 min	10 min
*γ*-Butyrolactone	2033	7644	10782	3820	9353	11068
*γ*-Hexalactone	20681	129160	155069	53359	143513	155513
*γ*-Heptalactone (IS)	38894	185530	230138	81918	209111	230259
Whiskey-lactone I	110605	364837	413042	199319	387667	413463
*γ*-Octalactone	111825	360337	407674	197209	384203	408474
Whiskey-lactone II	90756	320900	380063	183103	351145	380190
*γ*-Nonalactone	119260	374890	417670	212936	389701	418269
*γ*-Decalactone	106828	327823	363834	186756	339222	364786
3,4-Dimethylphenol (IS)	444709	999600	1090969	490860	1032004	1092175
*δ*-Decalactone	16550	130347	175598	49251	138751	175672
*γ*-Undecalactone	135335	367100	412196	217489	381118	411959

**Table 5:** O.O, slope, *R* ^2^, and linear range of lactones (*n* = 8).

Compound	Intercept	Slope	*R* ^2^	Linear range (*μ*g/L)
*γ*-Butyrolactone	0.001 ± 0.001	0.110 ± 0.001	0.999	0.17–60.26*
*γ*-Hexalactone	0.003 ± 0.002	1.445 ± 0.009	0.999	11–609
Whiskey-lactone I	0.003 ± 0.002	3.425 ± 0.003	0.999	1–401
*γ*-Octalactone	0.003 ± 0.001	3.418 ± 0.003	0.999	1–21
Whiskey-lactone II	0.005 ± 0.002	3.272 ± 0.002	0.999	1–401
*γ*-Nonalactone	0.004 ± 0.001	3.665 ± 0.003	0.997	2–206
*γ*-Decalactone	0.001 ± 0.001	3.294 ± 0.002	0.997	1–20
*δ*-Decalactone	0.001 ± 0.001	0.311 ± 0.001	0.997	4–517
*γ*-Undecalactone	0.004 ± 0.001	3.724 ± 0.003	0.999	1–15

*mg/L.

**Table 6:** LOD, repeatability, and reproducibility of method [36–38].

Compound	LOD (*μ*g/L)	LOQ (*μ*g/L)	Odor threshold (*μ*g/L)	Repeatability RSD (%)	Reproducibility RSD (%)
*γ*-Butyrolactone	170.97	569.93	35000	0.63	1.93
*γ*-Hexalactone	10.51	35.04	359000	2.98	2.57
Whiskey-lactone I	0.97	3.24	790	3.54	4.41
*γ*-Octalactone	1.19	3.98	7	4.31	3.96
Whiskey-lactone II	0.60	2.02	67	4.13	5.25
*γ*-Nonalactone	2.11	7.05	30	3.24	2.73
*γ*-Decalactone	0.86	2.89	88	2.50	2.78
*δ*-Decalactone	4.17	13.92	386	3.19	4.56
*γ*-Undecalactone	0.63	2.10	60	2.88	4.45

**Table 7:** Mean (%) and RSD (%) of recoveries.

Compound	Low level				High Level			
	Mean	Mean	RSD	RSD	Mean	Mean	RSD	RSD
	White	Red	White	Red	White	Red	White	Red
*γ*-Butyrolactone	189.1	191.9	1.45	2.56	189.4	198.6	4.23	0.14
*γ*-Hexalactone	88.1	86.7	3.05	5.62	88.6	88.5	1.80	0.77
Whiskey-lactone I	144.3	145.2	9.11	1.98	144.2	143.1	1.05	1.46
*γ*-Octalactone	76.8	81.3	2.06	3.21	78.1	80.1	0.65	2.10
Whiskey-lactone II	76.8	80.1	1.36	8.15	74.7	76.9	1.26	1.65
*γ*-Nonalactone	93.2	101.6	3.73	1.79	94.2	107.0	1.26	1.05
*γ*-Decalactone	136.5	142.8	0.96	3.45	137.5	147.4	2.33	0.38
*δ*-Decalactone	109.3	108.2	1.79	4.58	108.5	109.1	1.27	2.01
*γ*-Undecalactone	112.0	116.5	0.99	2.10	111.0	112.6	1.31	1.17

**Table 8:** Mean of recoveries and RSD.

Compound	Mean (%)	RSD (%)
*γ*-Butyrolactone	192.3	3.01
*γ*-Hexalactone	88.0	2.98
Whiskey-lactone I	144.2	4.09
*γ*-Octalactone	79.1	2.95
Whiskey-lactone II	77.1	4.52
*γ*-Nonalactone	99.0	6.23
*γ*-Decalactone	141.1	3.72
*δ*-Decalactone	108.8	2.37
*γ*-Undecalactone	113.0	2.27

**Table 9:** Concentration mean and SD (*μ*g/L).

Compound	White wines^1^ (*n* = 35)		Rosé wines^2^ (*n* = 8)		Red wines^3^ (*n* = 29)		Significative differences (*P* < 0.05)
	Mean	SD	Mean	SD	Mean	SD	
*γ*-Butyrolactone	26287	8478	25940	6553	32652	6403	1–3, 2-3
*γ*-Hexalactone	200	89	202	72	211	67	—
Whiskey-lactone I	10.60	14.38	5.44	2.20	45.73	34.42	1–3, 2-3
*γ*-Octalactone	5.41	2.64	5.63	2.20	6.95	3.13	—
Whiskey-lactone II	20.31	50.46	d-nq	—	138.44	111.13	1–3, 2-3
*γ*-Nonalactone	14.77	7.39	19.98	5.64	42.09	27.45	1–3, 2-3
*γ*-Decalactone	d-nq	—	4.16	2.91	3.85	2.65	1-2, 1–3
*δ*-Decalactone	157	72	149	37	228	96	1–3, 2-3
*γ*-Undecalactone	nd	—	d-nq	—	d-nq	—	—

nd: not detected; d-nq: detected not quantified.
